# Advanced Gene Therapy Strategies for the Repair of ACL Injuries

**DOI:** 10.3390/ijms232214467

**Published:** 2022-11-21

**Authors:** Mahnaz Amini, Jagadeesh K. Venkatesan, Wei Liu, Amélie Leroux, Tuan Ngoc Nguyen, Henning Madry, Véronique Migonney, Magali Cucchiarini

**Affiliations:** 1 Center of Experimental Orthopaedics, Saarland University Medical Center, Kirrbergerstr. Bldg 37, D-66421 Homburg, Germany; 2 Laboratoire CSPBAT UMR CNRS 7244, Université Sorbonne Paris Nord, Avenue JB Clément, 93430 Villetaneuse, France

**Keywords:** ACL, injuries, tissue engineering, gene therapy, biomaterial-guided gene transfer

## Abstract

The anterior cruciate ligament (ACL), the principal ligament for stabilization of the knee, is highly predisposed to injury in the human population. As a result of its poor intrinsic healing capacities, surgical intervention is generally necessary to repair ACL lesions, yet the outcomes are never fully satisfactory in terms of long-lasting, complete, and safe repair. Gene therapy, based on the transfer of therapeutic genetic sequences via a gene vector, is a potent tool to durably and adeptly enhance the processes of ACL repair and has been reported for its workability in various experimental models relevant to ACL injuries in vitro, in situ, and in vivo. As critical hurdles to the effective and safe translation of gene therapy for clinical applications still remain, including physiological barriers and host immune responses, biomaterial-guided gene therapy inspired by drug delivery systems has been further developed to protect and improve the classical procedures of gene transfer in the future treatment of ACL injuries in patients, as critically presented here.

## 1. Introduction

Injuries in the anterior cruciate ligament (ACL) of the knee are common, representing an important socioeconomical burden as they may occur both in young individuals following sport activities and in the aging population by chronic degeneration and result in diminished musculoskeletal functions and potentially leading to osteoarthritis [1,2,3]. Since the ACL has a slow and limited intrinsic ability for self-healing [2,4,5,6,7,8,9,10,11], various clinical options have been developed to enhance the reparative activities in sites of ACL injury, including conservative treatments and surgical interventions such as ACL reconstruction [11,12,13,14,15,16,17,18,19,20,21,22] using natural grafts (auto-/allografts) and synthetic materials [22,23,24,25,26,27,28,29]. Yet, none of these approaches afford the long-lasting healing of ACL lesions with original structural and mechanical features, and they can be associated with notable complications including pain, contractures, weakness, failure, re-ruptures, premature osteoarthritis, infections, and detrimental body responses [17,18,23,30,31,32,33,34,35,36,37,38,39,40,41].

To address these issues, novel therapeutic strategies were established to improve the mechanisms of ACL repair based on the further use of tissue engineering tools including adapted scaffolds (structural templates), cell-based material (reparative elements), bioreactors (environmental and loading control systems), and biological stimuli (regulatory factors and cues) [2,8,37,42,43,44,45,46,47,48,49,50,51,52,53,54,55,56,57,58,59,60,61,62,63,64,65,66,67,68,69,70,71,72], yet application of these systems in experimental settings was met with only limited success and did not allow for full and durable ACL repair.

In this regard, gene therapy may provide adapted tools for an improved, prolonged healing of ACL lesions by transfer of candidate genetic sequences with a gene carrier (vector) to extend the therapeutic activities of the gene products (growth and transcription factors, signaling molecules, therapeutic ribonucleic acids—RNAs) in sites of ACL injury [73,74,75,76,77,78,79,80,81,82,83,84,85,86,87,88,89,90,91,92,93]. However, clinical gene therapy is still hindered by different physiological barriers in the recipient, including physical obstacles (body fluids, dense joint extracellular matrices) and biological inhibitors (pH/enzymatic environment of the joint, neutralizing host immune responses, rate-limiting intracellular steps, gene dissemination to non-target locations) [83,94,95,96,97,98]. A very innovative approach to tackle these problems is to provide therapeutic gene vectors via biomaterial-guided delivery in sites of ACL injury as an off-the-shelf system, allowing for a safe, stabilized, and protected controlled release of the gene vehicles using biocompatible scaffolds (cargos) [82,83,87,90,99,100,101,102,103,104]. The goal of this work is, therefore, to provide an overview of the most advanced gene therapy procedures that aim at enhancing the repair of ACL injuries.

## 2. ACL: Basic Science, Clinical Questions

### 2.1. ACL Function and Structure

**Figure 1:**
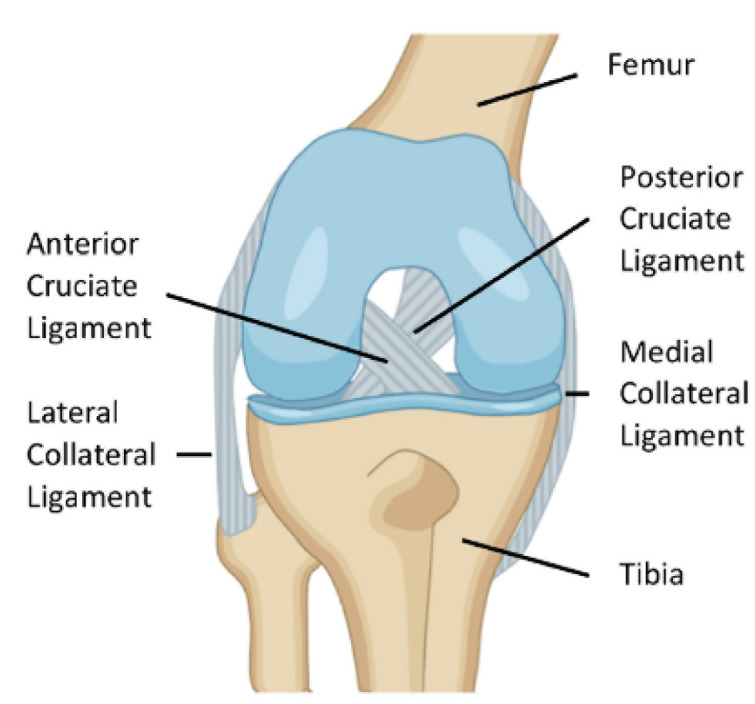
Knee key ligaments (anterior cruciate ligament—ACL, posterior cruciate ligament—PCL, medial collateral ligament—MCL, lateral collateral ligament—LCL).

The knee joint contains four ligaments that play key roles in kinematics and maintaining knee stability, connecting bones to each other, including two lateral ligaments that provide stability in the frontal plane and the anterior and posterior cruciate ligaments that support stability in the sagittal plane. Located in the center of the knee, the ACL forms part of the “central pivot” with the posterior cruciate ligament. The ACL is fundamental to connect the anterior part of the tibia to the posterior part of the femur, opposing forward displacement and excessive internal rotation of the tibia relative to the femur and stabilizing the knee during rotational movements [17,105,106,107] (Figure 1).

**Figure 2:**
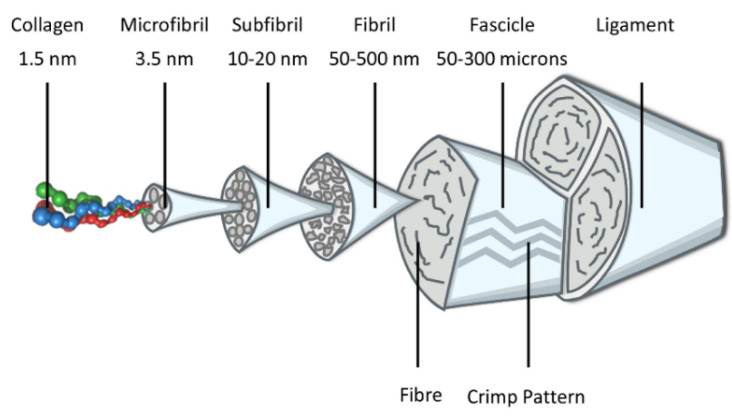
Schematic drawing of the multi-unit hierarchical structure of the ligament.

The ACL is a dense, cable-like tissue (27–32 mm in length, 10 mm in breadth, 4–10 mm in width, and 44.4–57.5 mm^2^ cross-sectional area) [46,105,108] that is highly organized, with an abundant extracellular matrix (ECM) mainly composed of collagens in fiber bundles (predominantly type-I collagen and types-III, -IV, -V, and -VI collagens; 70–80% dry weight) with elastin, fibronectin, thrombospondin, and proteoglycans (organization and lubrication of collagen fibril bundles) also surrounding ECM-producing cells (fibroblasts) in a hypocellular structure containing water (55–70%). The ACL has a hierarchical, sequentially assembled organization with increasing diameter and mechanical strength that includes collagen molecules (triple-helix polypeptide chains, <2 nm in diameter) that crosslink to form microfibrils (3.5 nm in diameter) that arrange themselves into subfibrils (10–20 nm in diameter) and then in fibrils (50–500 nm in diameter) that form fibers in fascicles (50–300 μm, with a crimp pattern repeated every 45–60 μm) that mutually crosslink to make a subfascicular unit running parallel to the long axis of the tissue, also containing proteoglycans and elastin and surrounded by a vascularized epiligament sheath to form the ligament [7,45,46,49,105,109,110,111,112] (Figure 2).

**Figure 3:**
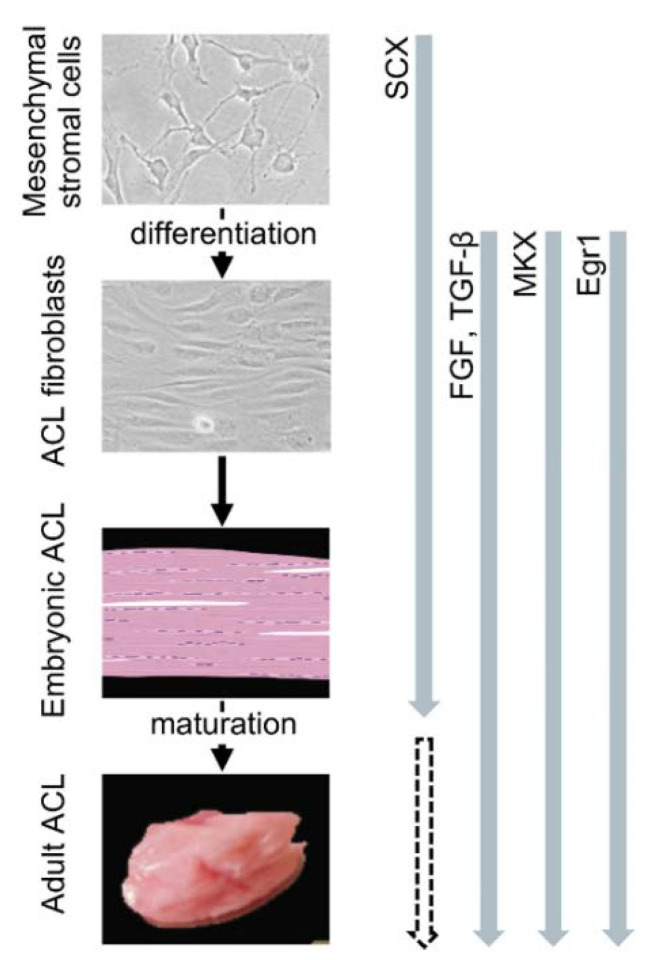
Expression patterns of critical transcription factors during the development of the ACL (SCX, scleraxis; FGF, fibroblast growth factor; TGF-β, transforming growth factor beta; MKX, Mohawk, Egr1, early growth response factor 1).

The cells in the ACL are interspersed between the collagen fibril bundles along the long axis, with a spindle-shaped form at an immature stage and a more elongated shape when they age. The ACL originates from the lateral plate mesoderm and its development is regulated by signaling from various growth factors (transforming growth factor beta—TGF-β—via TGFBR-receptor signaling and small mothers against decapentaplegic proteins—Smads; fibroblast growth factor—FGF—via FGFR-receptor signaling and extracellular signal-regulated kinase—ERK/mitogen-activated protein kinase—MAPK) and transcription factors (scleraxis—SCX—a basic helix-loop-helix factor; Mohawk—MKX—encoded by an atypical, three-amino-acid loop *homeobox* gene; early growth response factor 1—Egr1—a zinc finger transcription factor) that promote the differentiation of mesenchymal progenitor cells into ligament cells [11,105,113,114,115,116,117,118,119,120] (Figure 3).

### 2.2. Clinical Aspects: Pathology, Natural Healing, and Current Treatments

Ligament injuries are common, especially in the ACL (25–50% of knee ligamentous injuries; incidence of 1 per 3000 inhabitants per year in the United States and in Europe) [1]. ACL injuries are mainly derived from sport activities (65%) in the young population (70% of affected patients between 20–35 years old) and occur via chronic tissue degeneration in the aging population, both resulting in diminished musculoskeletal functions and potentially leading to osteoarthritis [1,35]. In the United States only, approximately 300.000 ACL reconstruction surgeries are performed annually, costing approximately USD 30 billion [2,3,10,60], clearly representing a significant public health problem.

The natural healing of ACL injuries is slow and inefficient, leading to the formation of scar tissue that does not naturally reproduce the original biological and mechanical properties of the tissue due to the limited intrinsic ability of the ACL to fully heal [2,4,5,6,7,8,10,11,13,14,15,16,46,54,121]. Three major cascade phases of ligament healing have been defined in response to injury, including (1) an inflammatory stage within the first days and weeks of injury (serous fluid accumulation, fragilization of the area, formation of a fibrin clot, invasion by monocytes/leukocytes/macrophages, debris removal, release of pro-angiogenic and proliferative growth factors and cytokines), (2) a proliferative stage by 8 weeks (blood vessel formation, fibroblast proliferation with collagen matrix production to fill up the injury), and (3) a remodeling phase between 1 and 2 years (decrease in cellularity, matrix realignment for adapted response to mechanical forces) [5,8]. The lack of proper ACL healing is due to the intra-articular conditions (synovial fluid, intra-articular movements) that hinder the stable formation of a fibrin/platelet scaffold (a prerequisite to primary healing), to an insufficient availability of reparative factors (growth factors and cytokines, wound filling compounds such as fibrinogen/fibronectin), to the lack of continuity between collagen fibers between the new and the old matrices leading to reduced tissue mechanical properties, and to the absence of vascularization of this ligament [2,5,8,10].

Due to the limited healing capacity of the ACL, a number of therapeutic options have been developed to restore its functions in the knee, including non-operative conservative treatments (immobilization/bracing/rest, exercise/physiotherapy, corticoid injection, etc.) and surgical procedures (ACL reconstruction, i.e., ligamentoplasty), while primary repair (suturing) was abandoned due to high rates of failure (40–100%) [2,11,12,17,18,19,20,21,22,23,37,60]. ACL reconstruction has been widely adopted as a standard procedure, especially for young patients, based on the use of both natural/biological grafts including autografts (patellar, hamstring, or quadriceps tendons) and allografts (frozen ligaments from cadavers), as well as commercially available synthetic or artificial materials and systems (substitutes, graft-augmented, and prosthetic devices) (Gore-Tex^®^—polytetrafluoroethylene—prosthesis; Lars^®^ ligament—terephthalic polyethylene polyester; Stryker-Dacron^®^ ligament—polyethylene terephthalate; Leeds-Keio^®^—polyester ethylene terephthalate; Kennedy ligament-augmentation device—LAD—braided polypropylene braid) [2,17,18,22,23,24,25,26,27,28,29,37,43,46,60,105,122]. While autografts display several advantages (good initial mechanical strength, adapted to promote cell proliferation and neotissue formation), their use is restricted by their limited availability, the necessity for second surgery for tissue harvest, the problem of donor site morbidity resulting in pain/contracture/weakness, and a lack of fully adequate mechanical strength over time [2,17,18,22,23,37,46,60]. Regarding allografts instead, second surgery for tissue harvest is not needed and there is no limit to the supply of graft tissue, generally allowing for good initial mechanical strength and supporting cell proliferation and neotissue formation. However, the use of allografts is associated with a possible transmission of diseases and infections, with potential host immunological foreign body responses, delayed biological integration, and a lack of fully adequate mechanical strength over time while their sterilization usually affects their mechanical properties [2,17,18,22,23,37,46,60]. Synthetic or artificial materials and systems have a number of advantages over natural grafts (availability, reproducibility, safety) but they may fail over time as they do not reliably reproduce the mechanical properties of the ACL, potentially leading to permanent deformation and degeneration upon repeated elongation with the creation of inflammatory debris in the joint [2,17,18,22,23,37,46,54,60]. While patients have benefited from ACL reconstruction, a significant percentage of them (20–25%) have unadapted and unsatisfactory outcomes and serious complications over time (7–10 years after surgery), including re-ruptures and premature osteoarthritic changes (50% incidence), both in young active patients and in elder individuals [9,30,31,32,33,34,36,38,39,40,41,123].

### 2.3. Experimental Treatments: Tissue Engineering and Biological Augmentation

As none of the current therapeutic interventions have, thus far, been capable of affording long-lasting ACL repair, other options have been envisaged to manage this critical clinical issue, including tissue engineering procedures and biological augmentation to replicate the natural environment of the ACL tissue and enhance its reparative activities based on the use of (1) biocompatible, biodegradable, minimally inflammatory, and mechanically adapted material scaffolds serving as structural and logistic templates, (2) cells and tissues providing reparative components, (3) bioreactors for loading and as control systems of the cellular environment, and (4) biological, biochemical, and biomechanical signals and nutrients as regulatory factors and cues [2,8,10,11,26,43,45,46,47,48,49,52,54,55,56,57,59,60,61,62,63,64,65,66,67,68,69,70,72,75,124,125,126,127,128,129,130,131,132,133,134,135,136,137,138,139,140,141,142,143,144,145,146,147,148,149].

Biomaterial scaffolds used for ACL repair include systems (solutions, gels, fibers, membranes, matrices, sponges) based on fibrin [150,151], hyaluronic acid (HA) [152,153,154,155], chitosan [156], collagen [44,157,158,159,160,161,162,163,164,165,166,167,168], silk [169,170,171,172,173,174,175,176,177,178,179,180,181,182,183], poly(glycolic acid) (PGA) [184,185,186], poly(lactic acid) (PLA) [186,187,188,189,190,191,192,193,194,195], poly(lactic-co-glycolic acid) (PLGA) [42,168,186,187,188,196,197,198], poly(caprolactone) (PCL) [199,200,201,202,203,204,205,206], polyurethane (PU) [168], oligo(poly(ethylene glycol) fumarate) (OPF) [207], poly(ethylene terephthalate) (PET) [208,209,210,211,212], and hybrid compounds such as collagen/glycosaminoglycan (GAG) [213,214], collagen/silk [215,216,217], collagen/poly(desaminotyrosyl-tyrosine dodecyl dodecanedioate) (p(DTD DD)) [218], silk/PLGA [219], chitosan/HA [220], chitosan/PLA [221], chitosan/PCL [222], PLA/PCL [223], PGA/PCL [184], and PLA/PLGA systems [224], with successful attempts at material functionalization for ACL repair using for instance poly(sodium styrene sulfonate) (polyNaSS) grafting [205,206,209,211] (Table 1).

Such scaffolds have been further employed in conjunction with cells upon seeding onto biomaterials [2,42,43,45,46,47,49,54,56,59,60,62,64,67,68,69,70,213] such as differentiated fibroblasts and tissue (ACL, skin) [159,160,166,168,178,182,184,186,188,189,194,196,200,205,206,209,214,221,222] and progenitor cells (bone marrow-derived mesenchymal stromal cells—MSCs, adipose-derived MSCs—ASCs) [44,153,163,165,167,169,170,171,172,173,174,175,176,177,179,181,183,190,196,197,198,199,201,207,219,223,224], or even exosomes (extracellular vesicles carrying proteins, lipids, and nucleic acids and critical mediators of cell-cell communication) prepared from MSCs [230,231], tested in bioreactors to provide adaption to a dynamic environment with biomechanical cues to improve tissue repair [44,50,51,54,55,62,63,68,146,170,199,207] with further biological augmentation [8,45,47,48,49,54,56,60,65,68,70,155] including growth factors (TGF-β, basic FGF—FGF-2, growth differentiation factor 5—GDF-5, platelet-derived growth factor—PDGF, epidermal growth factor—EGF, connective tissue growth factor—CTGF, insulin) [151,154,172,198,199,203,219] and platelet-rich plasma (PRP, a derivative of whole blood with supraphysiological concentrations of platelets, with fibrin, and allowing for the release of growth factors and other bioactive substances and having inhibitory effects on inflammatory cytokines) [52,53,57,58,59,61,64,65,66,69,70,71,72,137,148,164,232,233,234,235] used as a hydrogel in combination with collagen [225,226,227,228,229] (Table 1).

While the use of biomaterial scaffolds with or without augmentation (cells, biological/mechanical stimuli) proved beneficial at least to a certain point experimentally, these systems have again been unable to support long-lasting ACL repair, showing the critical need for improved treatment strategies to effectively manage this clinical problem. In this regard, gene therapy may provide powerful tools to adeptly heal sites of ACL injury.

## 3. Classical Gene Therapy for the Repair of ACL Injuries

Gene therapy is based on the transfer of genetic sequences in target cells, tissues/organs, and live organisms with gene vehicles as a means to prolong the therapeutic effects of one or various gene products relative to the application of recombinant factors with short half-lives [73,74,75,76,77,78,79,80,81,82,83,84,85,86,87,88,89,90,91,92,93,236,237]. Candidate factors for gene therapy may include growth and transcription factors, signaling molecules, as well as therapeutic RNAs that may be either suppressive (i.e., interfering) sequences such as oligodeoxyribonucleotide (ODNs), antisense RNAs, microRNAs (miRNAs), small interfering RNAs (siRNAs), short hairpin RNAs (shRNAs), and long non-coding RNAs (lncRNAs) or activating sequences such as messenger RNAs (mRNAs). Gene therapy can be performed either via a direct (in vivo) administration of the gene vector or via an indirect (ex vivo) supply of cells/grafts that are genetically modified in vitro prior to reimplantation in the recipient.

### 3.1. Gene Transfer Vectors

Gene vehicles include both nonviral and viral systems with specific characteristics that make them more adapted for in vivo or for ex vivo therapy (Table 2).

Nonviral vectors are non-replicative, non-immunogenic, safe systems without size limitation, but they exhibit a relatively low gene transfer efficiency for only very short periods of time (<40% for few weeks) since the genes being carried are kept as episomes that necessitate cell division for effective expression, making these vectors more suitable for ex vivo therapy [238,239].

Viral vectors that employ the ability of viruses to penetrate various cell types include adenoviral, herpes simplex viral (HSV), retro-l/lentiviral, and recombinant adeno-associated virus (rAAV) vectors. Adenoviral and HSV vectors are capable of directly modifying dividing and quiescent cells at elevated efficiencies (~100%), making them adapted for in vivo therapy, but they support only short-term (episomal) transgene expression (between some days to 1–2 weeks) while activating host immune responses [98,240,241,242]. Retroviral vectors have the ability to integrate in cellular genomes, allowing for long-term transgene expression, but they have low efficiencies (<20%), making them more suited for ex vivo therapy, and can only modify dividing cells, with possible insertional mutagenesis [243], while being also potentially immunogenic [98]. As an alternative, lentiviral vectors can also target quiescent cells, but they derive from the pathogenic human immunodeficiency virus (HIV) and may also lead to insertional mutagenesis [244]. rAAVs are small (~20 nm), safe vectors devoid of viral sequences and that are capable of modifying both dividing and quiescent cells at elevated efficiencies over extended periods of time (~100% for months to years) due their maintenance as stably expressed episomes [245,246], making them adapted for in vivo therapy, but they may raise immune responses, in particular by pre-existing neutralizing antibodies directed against the viral capsid proteins [98,247,248,249].

### 3.2. Candidate Therapeutic Factors

Therapeutic factors amenable to gene transfer to repair ACL injuries include growth factors, transcription factors, anti-inflammatory agents, matrix components, and signaling molecules.

A variety of polypeptide growth factors have been reported for their beneficial activities to repair ACL injuries. They include TGF-β [198,250,251,252,253,254,255,256,257,258], bone morphogenetic proteins (BMPs) and GDFs (BMP-2; GDF-5, i.e., BMP-14; GDF-6, i.e., BMP-13; GDF-7, i.e., BMP-12) [198,259,260,261,262], FGF-2 [251,255,256,262,263,264,265,266], insulin-like growth factors (IGF-I, IGF-II) [250,251,252,264], PDGF [251,252,256,258,260,264,265,267,268], EGF [251,252,254,265,269,270], hepatocyte growth factor (HGF) [260], CTGF [151], and vascular endothelial growth factor (VEGF) [271,272]. These factors play critical roles in stimulating cellular activities relevant for ACL repair such as cell migration and proliferation (TGF-β, GDFs, BMP-2, FGF-2, IGF-I, PDGF, EGF, HGF, CTGF, VEGF) [151,198,251,252,253,255,256,257,258,260,262,263,265,267,268,269,270,272], cell adhesion (GDFs, FGF-2, EGF) [262,269], matrix deposition (type-I/-III collagen, proteoglycans) (TGF-β, GDFs, FGF-2, IGF-I/-II, PDGF, EGF, CTGF) [151,198,250,252,254,255,256,258,259,261,262,266,268], angiogenesis (FGF-2, PDGF, VEGF) [266,268,271,272], and mechanical properties (stiffness) (TGF-β, FGF-2, IGF-I, PDGF, CTGF) [151,257,258,264,268].

Transcription factors have been also described for their advantageous properties for ACL repair [115,118,119,120,273], among which are SCX, regulating cell differentiation and matrix synthesis (type-I collagen, fibromodulin, tenomodulin—TNMD, decorin), especially in tendon progenitors [113,114,117,274,275,276,277,278,279], and MKX, essential for tendon/ligament differentiation by regulating the expression of matrix components (type-I collagen, fibromodulin, TNMD, decorin, lumican) [116,280,281,282,283].

Other components may be used as potential candidates for the repair of ACL lesions such as factors that may prevent the potential inflammation associated with injury [284,285,286,287,288,289,290,291,292,293,294,295] such as an anti-inflammatory IL-1 receptor antagonist (IL-1Ra) [295,296,297], soluble TNF-α receptor [296], IL-10 [298,299], or lysyl oxidase (LOX), an enzyme that oxidizes amino acid residues in collagens and elastin, allowing these compounds to bind to each other and to repair the extracellular matrix, and which can suppress inflammation in ACL cells [300]. Alternative candidates for ACL repair may also conceptually include matrix components such as collagens [301,302], TNMD [303,304,305,306], decorin [307,308,309], fibromodulin [310], biglycan [308,309], or others, as their presence is critical to the natural structure and functional integrity of the ACL. Other factors may also tentatively provide interesting candidates for ACL repair, such as those involved in signaling processes involved in ACL formation (wingless integrated—Wnt -/β-catenin, sonic hedgehog—SHH, FGF signaling via specific receptors, and ERK/MAPK, BMP/TGF-β/GDF signaling via specific receptors and Smads) [115,118,119,273,311,312].

### 3.3. Applications of Classical Gene Therapy for ACL Repair

Therapeutic gene therapy in the goal of ACL repair has been performed in relevant experimental models in cell culture in vitro, in tissue (explant) culture in situ, and in relevant animal models using both direct (in vivo) and indirect (ex vivo) gene transfer approaches.

In vitro and in situ (Table 3), the application of therapeutic genes has been reported using nonviral [313], adenoviral [282,314,315,316,317,318,319,320,321,322,323,324], retro-/lentiviral [275,283,314,315,325,326,327], and rAAV vectors [328].

These vectors were employed to deliver reporter genes (β-galactosidase—*lacZ*, green fluorescent protein—GFP, luciferase—*luc*) [313,314,315,317,318,328] and therapeutic sequences (TGF-β, BMP-2, BMP-12, BMP-13, FGF-2, IGF-I, PDGF, VEGF, SCX, MKX) [275,282,283,313,316,317,318,319,320,321,322,323,324,325,326,327,333] in rabbit, bovine, and human ACL cells and ACL explant (normal, experimentally torn) tissue [314,315,317,318,320,322,328] and in mouse, rat, rabbit, and human progenitor cells (MSCs, ACL-derived stem cells, perichondrial cells, induced pluripotent stem cells—iPSCs) [275,282,283,313,316,319,321,322,323,324,325,326,327,333]. Gene transfer allowed for an effective expression of the sequences being delivered for up to 2 days using nonviral vectors [313], one week using adenoviral vectors [282,314,315,316,317,318,319,320,321,323] or longer (2 weeks) if the modified cells where placed in hydrogel (type-I collagen) cultures [322], 12 weeks using retro-/lentiviral vectors upon cell selection [283,314,315,323,325,326,327,333], and at least one month using rAAV vectors [328]. Therapeutic gene transfer (TGF-β, BMP-12, BMP-13, FGF-2, IGF-I, VEGF) was capable of enhancing cell proliferation [317,318,320,322,328] using adenoviral vectors [317,318,320,322] for up to 2 weeks [317] and rAAV vectors for up to one month [328]. Therapeutic gene transfer (TGF-β, BMP-12, BMP-13, FGF-2, IGF-I, VEGF, SCX, MKX) also led to increased levels of matrix deposition and of specific marker expression (type-I/-III collagen, elastin, vimentin, fibromodulin, fibronectin—FN, tenascin, TNMD, decorin, lumican, biglycan, SCX, contractile alpha-smooth muscle actin—α-SMA) [275,282,283,317,318,320,322,324,327,328] using adenoviral vectors [282,317,318,320,324] for up to 3 weeks [283,317,322], retro-/lentiviral vectors over time upon cell selection [275,283,327], and rAAV vectors for up to one month [282]. Strikingly, rAAV-mediated gene delivery of FGF-2 over time (one month) was able to heal experimentally created human ACL lesions in situ via the enhanced expression of contractile α-SMA and of the ligament-specific SCX transcription factor and via increased collagen deposition [328]. Therapeutic RNAs have been also applied in vitro [329,330,334,335,336] and in situ [331], based on the delivery of ODNs (decorin, type V procollagen α1 chain) [329], miRNAs (Rho-associated coiled-coil protein kinase 1—ROCK1) [336], shRNAs (decorin) [335], lncRNAs (H19 lncRNA involved in TGF-β signaling) [334], and mRNAs (BMP-7) [331] using nonviral [329,330,331] and retro-/lentiviral vectors [334,335,336] to target rat, rabbit, and human ACL (and tendon) cells and explant tissue [329,330,331,335] and rat and human progenitor cells (tendon-derived stem cells) [334,336], allowing to suppress the expression of each specific marker while improving the ligamentous/tenogenic phenotype for up to one week [329,330,331,334,335,336].

Direct (in vivo) administration of therapeutic genes in animal models (Table 4) has been performed using nonviral [337,338,339] and adenoviral vectors [340,341,342] to deliver reporter genes (*lacZ*) [337,339,340,341] and therapeutic sequences (BMP-13, PDGF) [338,342] in experimentally created ligamentous lesions in rats [337,338,339,342] and in rabbits [340,341], allowing for the effective expression of the sequences being delivered for up to 2 weeks using nonviral vectors [337,339] and 6 weeks using adenoviral vectors at very high doses [340].

Therapeutic gene transfer (BMP-13, PDGF) was capable of enhancing type-I collagen deposition for 4 weeks in injured rat ligaments using nonviral vectors (PDGF) [338] and of promoting neoligament formation with increased levels of cell proliferation and collagen deposition for up to 14 weeks in a similar experimental model using adenoviral vectors (BMP-13) [342]. Therapeutic RNAs have been also directly applied in vivo [331,343,344,345], based on the delivery of ODNs (decorin) [343,345] and mRNAs (BMP-7, FGF-2) [331,344] using nonviral vectors [331,343,345] or only RNA solutions [344] in experimentally created ligamentous lesions in vivo in rats [331,344], in rabbits [343,345], and in sheep [331], allowing to suppress the expression of each specific marker while enhancing collagen deposition, mechanical stiffness, and healing for up to 6 weeks [331,343,344,345].

Indirect (ex vivo) application of therapeutic genes in animal models (Table 5) has been performed using nonviral [346], adenoviral [316,341,347,348], and retro-/lentiviral vectors [325,326,333,340] to deliver reporter genes (*lacZ*, GFP) [340,341,346,347] and therapeutic sequences (TGF-β, BMP-2, BMP-6, BMP-12, PDGF, VEGF) [316,325,333,346,347,348] as a means to modify rabbit ACL cells [340,341], rabbit and minipig ACL and tendon tissue grafts [346,347], and mouse, rat, rabbit, and human progenitor cells (MSCs, ACL-derived stem cells) [316,325,326,333,348] prior to applying them to ectopic models (muscle injection) in mice [316] or to experimentally created ligamentous lesions or for ACL replacement and repair in vivo in rats [325,326], in rabbits [333,340,341,347,348], and in minipigs [346], allowing for the effective expression of the sequences being delivered for up to 2 weeks using nonviral vectors [346] or adenoviral vectors [341,347] and 10 days using retro-/lentiviral vectors [340].

Therapeutic gene transfer (TGF-β, BMP-2, BMP-6, BMP-12, PDGF, VEGF) was capable of enhancing matrix deposition and specific marker expression (type-I/-III collagen, α-SMA) and promoting neoligament formation with increased levels of collagen deposition and improved mechanical properties in injured mouse, rat, rabbit, and minipig ligaments for up to 8 weeks using nonviral vectors (BMP-6) [346], 26 weeks using adenoviral vectors (TGF-β, BMP-2, BMP-12, VEGF) [316,347,348], and 12 weeks using retro-/lentiviral vectors (BMP-2, PDGF, VEGF) [325,326,333]. Therapeutic RNAs have been also indirectly applied in vivo [334,335], based on the delivery of shRNAs (decorin) [335] and lncRNAs (H19 lncRNA involved in TGF-β signaling) [334] using retro-/lentiviral vectors [334,335] as a means to modify rat tendon cells [335] and human progenitor cells (tendon-derived stem cells) [334] prior to applying them to experimentally created tendon lesions in vivo in mice [334] and in rats [335], allowing to enhance matrix deposition and specific marker expression (type-I collagen, TNMD, decorin) for up to 4 weeks [334,335].

### 3.4. Limitations of Classical Gene Therapy for ACL Repair

While experimental work showed the potential benefits of classical gene therapy for ACL repair, a number of critical limitations need to be carefully addressed before initiating relevant approaches for translational regenerative therapy in the field of clinically adapted repair in patients.

ACL gene therapy may be first hindered by pre-existing physical obstacles and biological barriers to effective and safe therapeutic gene transfer, including the presence of inhibitory factors in the joint (body fluids, i.e., synovial fluid; clinical compounds, i.e., heparin), the local pH and/or enzymatic environment, and the dense extracellular matrix of the tissue itself that may impair the penetration of the vectors before reaching the target cells [82,83,85,87,90,349]. Another impairment is associated with the existence of innate and/or adaptive responses from the immune system of the recipient (antibodies, cellular helper, and cytotoxic T cells) that may be directed against the viral particles and/or the transgene sequence (neutralization processes) [98,247,350,351,352,353]. Various cell-associated steps may also limit the rate and occurrence of therapeutic gene expression such as the effective uptake of the gene vector (presence/amount of the specific cell membrane receptor/co-receptor to a particular vector type), its successful internalization and transport in the cell (endosomal escape and nuclear entry), as well as its active processing (levels of adapted cell activity, transgenic genome conversion) [94,95,96,241,354,355,356]. Other challenges to address also involve inherent features of the vectors that may potentially affect the efficacy of the therapeutic treatment (vector dissemination to non-target sites in the joint, toxicity of viral vector proteins, genotoxicity upon a possible transgene integration in the host genome, transformation risk) [97].

A variety of strategies have been developed to tackle such issues, including (1) the use of alternative routes of vector injection, vector doses, clinical components (passive hirudin versus inhibitory heparin), immunosuppressive agents, and/or alternative (viral) vector serotypes, (2) the modification/masking of viral particles to evade host immune responses (chemical conjugation with polyethylene glycol—PEG, tropism/viral capsid tailoring by inclusion of substitute peptide sequences/epitopes, viral capsid engineering using chimeric/hybrid/mosaic/shuffled/variant/mutant/decoy vectors or virus-like particles—VLPs, vector-like microvesicles, vexosomes), and (3) the modification of the vector genome to circumvent the rate-limiting steps of its intracellular processing (tissue-specific/activatable/disease-responsive promoters, hybrid/self-complementary vectors, optimized coding/noncoding sequences in the transgene cassettes, artificial chromosomes) [82,83,85,87,90,96,245,357,358,359,360,361]. Still, even though such approaches led to some improvements in the efficacy of gene transfer, they remain complex (capsid/vector genome modification and engineering) or do not allow for sufficient and adapted therapeutic effects and outcomes, especially in vivo, while the variability of the host immune responses between patients has not been carefully taken into account with any of these techniques. Overall, these observations show the critical need to develop novel, suitable tools to address such challenges for clinical gene therapy, in particular for human ACL repair.

## 4. Biomaterial-Guided Gene Therapy for the Repair of ACL Injuries

The administration of therapeutic gene vectors using biomaterials already employed in tissue engineering research is an attractive approach to tackle the current obstacles of clinical human gene therapy and regenerative medicine [82,83,85,86,87,90,93,102,103,104,362,363] and might be applied in the goal of human ACL repair [90,91].

### 4.1. Principles

Biomaterial-guided gene therapy is a groundbreaking, convenient therapeutic concept based on the controlled delivery of gene transfer vectors from biocompatible scaffolds (hydrogels, solid scaffolds) originating from approaches developed to apply drugs and recombinant agents with such systems in human medicine [139,364,365,366,367,368,369]. Biomaterial-guided gene therapy instead combines the application of gene vehicles using materials (cargos) that may mimic the properties of the ACL tissue while strengthening it [99,100,101,370,371,372,373,374,375,376,377,378,379,380,381,382] in order to allow for a spatiotemporal, safe expression of therapeutic genetic sequences in the recipient via an off-the-shelf, cell-free (patient-independent) compound. Biomaterials may be derived from natural (biocompatible, biodegradable) or synthetic (reproducible) components, or both, to either encapsulate gene vectors to achieve a polymeric vector release (gradient release by degradation of the polymer loaded with the vectors during scaffold formation for hydrogels) or to immobilize the gene vectors to achieve a substrate-mediated release (release of the vectors incorporated at the surface of preformed scaffolds for solid scaffolds) [383]. Gene therapy guided via controlled release from biomaterials may allow for the stabilization of the gene vectors against degradation, to enhance the residence time of the therapeutic genes being carried and the effects of their products, to minimize vector dissemination to nontarget sites and the vector doses needed to be applied in patients, and to mask potentially immunogenic viral particle epitopes and protect the vectors from host immune responses [82,83,85,87,90,102].

### 4.2. Applications of Biomaterial-Guided Gene Therapy for ACL Repair

In vitro and in situ (Table 6), biomaterial-guided application of therapeutic genes has been reported using nonviral [384] and adenoviral vectors [317,318] delivered via type-I collagen hydrogels [317,318] and PLGA nanospheres [384]. These vectors were employed to transfer reporter genes (GFP) [317,318,384] and therapeutic sequences (TGF-β, IGF-I) [317,318] in bovine ACL cells [317], chicken tendon cells [384], and bovine and human ACL explant (experimentally torn) tissue [317,318].

Gene transfer allowed for an effective expression of the sequences being delivered for up to 3 weeks using nonviral vectors [384] and 4 weeks using adenoviral vectors [317,318]. Therapeutic gene transfer (TGF-β, IGF-I) was capable of enhancing cell proliferation using adenoviral vectors for up to 3 weeks [318] and the levels of matrix deposition (type-I/-III collagen) using adenoviral vectors for up to 4 weeks [317]. Strikingly, adenoviral-mediated gene delivery of TGF-β was further able to heal experimentally created bovine ACL lesions over a prolonged period of time (4 weeks) in situ [317]. Therapeutic RNAs have been also applied in vitro (Table 6) via delivery of miRNAs (TGF-β) using nonviral vectors delivered via PLGA nanospheres to target chicken tendon cells, allowing for the suppression of the expression of TGF-β for up to 3 weeks [384].

Biomaterial-guided application of therapeutic genes in animal models (Table 6) has been performed using nonviral [385], adenoviral [319], and rAAV vectors [387,388] to deliver reporter genes (*luc*) [388] and therapeutic sequences (TGF-β, GDF-5, BMP-12) [319,385,387,388] as a means to modify mouse [387,388] and rabbit tendon tissue grafts [385] and rat muscle tissue grafts [319] prior to applying them to experimentally created tendon and ligamentous lesions for tissue replacement and repair in vivo in mice [387,388], in rats [319], and in rabbits [385], allowing for the effective expression of the sequences being delivered for up to 3 weeks [387]. Therapeutic gene transfer (TGF-β, GDF-5, BMP-12) was capable of promoting wound healing with increased levels of collagen deposition and improved mechanical properties in injured mouse, rat, and rabbit tendons and ligaments for up to 6 months using nonviral vectors (TGF-β) [385], 4 weeks using adenoviral vectors (BMP-12) [319], and 3 weeks using rAAV vectors (GDF-5) [387,388]. Therapeutic RNAs have been also applied via biomaterials in animal models (Table 6) [335,384,386,389,390,391], based on the delivery of shRNAs (decorin) [335] and miRNAs (TGF-β, angiogenic miR-210) [384,386,389,390,391] using nonviral [384,386] and lentiviral vectors [335] or only as RNA solutions [389,390,391] delivered via rat tendon tissue grafts [335], type-I collagen hydrogels [389,390,391], PLGA nanospheres [384], and 3D-bioprinted composite (PCL, polydopamine—PDA—nanoparticles—NPs, gelatin, HA, alginate) scaffolds [386] in experimentally created tendon and ligamentous lesions for tissue replacement and repair in vivo in rats [335,389,390,391] and in chickens [384,386], allowing to suppress the expression of the specific marker (TGF-β) for up to 6 weeks [384] while enhancing matrix deposition and specific marker expression (type-I collagen, VEGF), mechanical stiffness, and healing for up to 12 weeks [335,384,386,389,390,391].

## 5. Conclusions

To address the unsolved problem of achieving long-lasting, safe, and mechanically competent ACL repair in patients, as none of the currently available clinical options (ACL reconstruction, engineering, augmentation) can competently afford it thus far, advanced strategies were developed to improve the intrinsic mechanisms of tissue repair in prevalent ACL injuries based on gene therapy procedures using durable therapeutic gene transfer (growth/transcription factors, anti-inflammatory agents, matrix components, signaling molecules, therapeutic RNAs) in vectors in experimental systems in vitro and in situ and relevant animal models in vivo via classical gene transfer methods (direct gene vector administration, indirect implantation of genetically modified cells and tissues). While such experimental approaches met undeniable success, they may be limited by the existence of physical and biological barriers in patients such as the joint environment (inhibitory soluble factors, dense extracellular matrix, immune host responses) and rate-limiting steps to effective therapeutic gene expression (ACL cell-associated rate-limiting steps, vector dissemination and toxicity). Besides active work using complex vector engineering techniques, a more convenient strategy, namely biomaterial-guided gene therapy, has emerged to tackle these issues via the application of biocompatible materials as cargos for therapeutic gene vectors, allowing for their spatiotemporal, safe, and prolonged controlled release and expression in the recipient while mimicking the properties of the ACL tissue and strengthening it. With promising results reported in experimental systems in vitro and in situ and in relevant animal models in vivo using this highly innovative procedure, work is now needed to confirm its workability in large preclinical animal models before envisaging a possible translation in individuals that first requires approval by regulatory organizations [83,392,393,394]. With this in mind, it still remains to be determined which biomaterial (type, production method possibly including three-dimensional—3D—bioprinting to mimic the structural features of the ACL) [395,396,397,398,399], vector (class, dose), and gene (single sequence or combination of genes, potential use of direct genome editing such as the clustered regularly interspaced short palindromic repeats (CRISPR)/CRISPR-associated (Cas) system) [400,401,402,403,404,405,406,407,408,409] will be the most appropriate to effectively and permanently heal ACL lesions in order to be accessible to the patients in a near future.

## Figures and Tables

**Table 1:** Applications of tissue engineering for ACL repair.

Scaffolds	Cells	Factors	Bioreactors	Effects	Refs.
fibrin	-	-	-	improved ACL repair (goats)	[150]
		CTGF	-	improved ACL repair (rabbits)	[151]
HA	-	-	-	improved ACL repair (rabbits, patients)	[152,155]
		TGF-β, FGF-2, PDGF, insulin	-	higher cell outgrowth in ACL explants	[154]
	MSCs	-	-	higher cell growth, differentiation	[153]
chitosan	ACL fibroblasts	-	-	higher cell adhesion, differentiation	[156]
collagen	-	-	-	improved ACL repair (rabbits, pigs)	[157,158,161,162,164]
	ACL, skin fibroblasts	-	-	higher cell growth, differentiation	[159,160,166,168]
	MSCs	-	+	higher cell growth, differentiation, improved ACL repair (rabbits)	[44,163,167]
	ASCs	-	-	higher cell growth, differentiation	[165]
silk	-	-	-	improved ACL repair (pigs, goats)	[179,180]
	ACL, skin fibroblasts	-	-	higher cell growth, differentiation, improved ACL repair (dogs)	[171,178,182]
	MSCs	-	-	higher cell growth, differentiation, improved ACL repair (sheep)	[169,171,173,174,175,176,177,179,181,183]
			+	higher cell growth, differentiation	[170]
		TGF-β, FGF-2, EGF	-	higher cell growth, differentiation	[172]
PGA	-	-	-	improved ACL repair (dogs)	[185]
	ACL fibroblasts	-	-	higher cell growth, differentiation	[184,186]
PLA	ACL fibroblasts	-	-	higher cell growth, differentiation	[186,188,189,194]
	MSCs	-	-	higher cell growth, differentiation	[190,194]
PLGA	ACL, skin fibroblasts	-	-	higher cell growth, differentiation	[168,186,188,196]
	MSCs	-	-	higher cell growth, differentiation	[196,197]
		TGF-β, GDF-5	-	higher cell growth, differentiation	[198]
PCL	-	FGF-2	-	improved ACL repair (rats)	[203]
	ACL fibroblasts	-	-	higher cell growth, differentiation	[200,204,205,206]
	MSCs	-	+	higher cell growth, differentiation	[201]
		FGF-2	+	higher cell growth, differentiation	[199]
PU	ACL fibroblasts	-	-	higher cell growth, differentiation	[168]
OPF	MSCs		+	higher cell growth, differentiation	[207]
PET	-	-	-	tissue integration (sheep)	[211]
	ACL fibroblasts	-	-	higher cell growth, differentiation	[209]
collagen/GAG	ACL explants	-	-	higher cell migration, differentiation	[213,214]
collagen/silk	-	-	-	improved ACL repair (rabbits)	[215,216,217]
collagen/p(DTD DD)	-	-	-	improved ACL repair (sheep)	[218]
collagen/PRP	-	-	-	improved ACL repair (dogs, pigs)	[164,225,226,227,228]
	ACL fibroblasts	-	-	higher cell growth, differentiation	[229]
silk/PLGA	MSCs	FGF-2	-	higher cell growth, differentiation	[219]
chitosan/HA	-	-	-	improved ACL repair (rats)	[220]
chitosan/PLA	ACL fibroblasts	-	-	higher cell growth, differentiation	[221]
chitosan/PCL	ACL fibroblasts	-	-	higher cell growth, differentiation	[222]
PLA/PCL	MSCs	-	-	higher cell growth, differentiation	[223]
PGA/PCL	ACL fibroblasts	-	-	higher cell growth, differentiation	[184]
PLA/PLGA	MSCs	-	-	improved ACL repair (rabbits)	[224]

Abbreviations: HA, hyaluronic acid; PGA, poly(glycolic acid); PLA, poly(lactic acid); PLGA, poly(lactic-co-glycolic acid); PCL, poly(caprolactone); PU, polyurethane; OPF, oligo(poly(ethylene glycol) fumarate); PET, poly(ethylene terephthalate); GAG, glycosaminoglycan; p(DTD DD), poly(desaminotyrosyl-tyrosine dodecyl dodecanedioate); PRP, platelet-rich plasma; MSCs, bone marrow-derived mesenchymal stromal cells; ACL, anterior cruciate ligament; ASCs, adipose-derived MSCs; CTGF, connective tissue growth factor; TGF-β, transforming growth factor beta; FGF-2, basic fibroblast growth factor; PDGF, platelet-derived growth factor; EGF, epidermal growth factor; GDF-5, growth differentiation factor 5.

**Table 2:** Gene transfer vectors.

Vectors		Integration	Advantages	Limitations
Type	Class			
nonviral vectors		no	. large capacity . cost effectiveness . no infectiosity/replication . no toxicity/immunogenicity	low efficacy short-term expression only dividing cells
viral vectors	adenoviral	no	. large capacity . high efficacy . dividing/quiescent cells	. short-term expression . toxicity/immunogenicity
	HSV	no	. large capacity . high efficacy . dividing/quiescent cells	. short-term expression . toxicity/immunogenicity
	retroviral	yes	. large capacity . long-term expression	. low efficacy without selection . only dividing cells . toxicity/immunogenicity . potential insertional mutagenesis
	lentiviral	yes	. large capacity . dividing/quiescent cells . long-term expression	. low efficacy without selection . toxicity/immunogenicity . potential insertional mutagenesis . HIV material
	rAAV	no	. high efficacy . dividing/quiescent cells . long-term expression	. small capacity . possible immunogenicity . potential insertional mutagenesis

Abbreviations: HSV, herpes simplex viral vector; rAAV, recombinant adeno-associated viral vectors; HIV, human immunodeficiency virus.

**Table 3:** Applications of classical gene therapy for ACL repair in vitro and in situ.

Vectors	Cell Targets	Genes	Effects	Refs.
nonviral vectors	rabbit ligament cells	ODN (decorin)	suppression of decorin expression (one day)	[329]
	human tendon cells	ODN (TVPα1)	suppression of TVPα1 expression (one day)	[330]
	horse, bovine, sheep, pig, rat tendons	mRNA (*lacZ*, *luc*, BMP-7)	effective gene expression (one day)	[331]
	rabbit perichondrial cells	*lacZ*, TGF-β	effective gene expression (2 days)	[313]
adenoviral vectors	rabbit ACL cells	*lacZ*, TGF-β, VEGF	effective gene expression, high DNA, type-I/-III collagen, FN contents (3 days)	[314,315,320]
	bovine ACL cells	GFP, *luc*, TGF-β (type-I collagen gel)	effective gene expression (6 days), high DNA and type-I/-III collagen contents (3 weeks)	[317]
	human ACL cells	GFP, *luc*, BMP-12, -13, IGF-I (type-I collagen gel)	effective gene expression, high DNA, type-I/-III collagen, tenascin, TNMD, elastin, vimentin, decorin, FN, biglycan, SCX contents (3 weeks)	[318,322]
	murine MSC line	BMP-12	effective gene expression (5 days)	[316,332]
	rat MSCs	SCX	effective gene expression (one day)	[323]
	rabbit MSCs	TGF-β, VEGF	effective gene expression (2 days)	[321]
	human MSCs	BMP-12, -13 (type-I collagen gel)	effective gene expression (2 weeks), high DNA, type-III collagen, tenascin, TNMD, elastin, vimentin, decorin, FN, biglycan, SCX contents (3 weeks)	[322,324]
		SCX, MKX	effective gene expression, high type-I collagen, tenascin, TNMD contents (one week)	[282]
retroviral vectors	rabbit ACL cells	*lacZ*	effective gene expression (one week)	[314,315]
	murine MSC line	MKX	high type-I collagen, decorin contents (5 days)	[283]
	rabbit MSCs	PDGF	effective gene expression (12 weeks)	[333]
	human tendon-derived stem cells	lncRNA (H19)	enhanced tenogenic differentiation (one week)	[334]
lentiviral vectors	rat tendon cells	shRNA (decorin)	suppression of decorin expression (3 days)	[335]
	rat ACL-derived stem cells	VEGF	effective gene expression (2 days)	[325]
	rat tendon-derived stem cells	miRNA (ROCK1)	high tenogenic differentiation (one week)	[336]
	human MSCs	SCX	effective gene expression, high type-I collagen, TNMD, decorin, FN, fibromodulin, lumican, α-SMA contents (cell selection)	[275]
	human ACL-derived stem cells	BMP-2	effective gene expression (3 days)	[326]
	human iPSCs	MKX	high type-III collagen, decorin, fibromodulin, SCX contents (cell selection)	[327]
rAAV vectors	human ACL cells, explants (normal, torn)	*lacZ*, FGF-2	effective gene expression, high DNA, type-I/-III collagen, SCX, α-SMA, NF-κB contents (one month)	[328]

Abbreviations: rAAV, recombinant adeno-associated viral vectors; ACL, anterior cruciate ligament; MSCs, mesenchymal stromal cells; iPSCs, induced pluripotent stem cells; ODN, oligodeoxyribonucleotide; TVPα1, type V procollagen α1 chain; mRNA, messenger ribonucleic acid; *lacZ*, β-galactosidase; *luc*, luciferase; BMP, bone morphogenetic protein; TGF-β, transforming growth factor beta; VEGF, vascular endothelial growth factor; GFP, green fluorescent protein; IGF-I, insulin-like growth factor I; SCX, scleraxis; MKX, Mohawk; PDGF, platelet-derived growth factor; lncRNA, long non-coding RNA (H19 involved in TGF-β signaling); shRNA, short hairpin RNA; miRNA, microRNA; ROCK1, Rho-associated coiled-coil protein kinase 1; FGF-2, basic fibroblast growth factor; DNA, deoxyribonucleic acid; TNMD; tenomodulin; FN, fibronectin; α-SMA, alpha-smooth muscle actin; NF-κB, nuclear factor kappa B.

**Table 4:** Applications of classical gene therapy for direct ACL repair in vivo.

Vectors	Animal Models	Genes	Effects	Refs.
nonviral vectors	rat ligament lesion	*lacZ*, PDGF	effective gene expression, high type-I collagen deposition (4 weeks)	[337,338,339]
		ODN (decorin)	suppression of decorin expression (4 weeks), high type-I collagen deposition with stronger mechanical properties (6 weeks)	[338,343]
	rat, sheep tendon injury	mRNA (BMP-7)	effective gene expression, high type-III collagen deposition (one week)	[331]
adenoviral vectors	rat ligament lesion	BMP-13	high collagen deposition, neoligament formation (14 weeks)	[342]
	rabbit ligament lesion	*lacZ*	effective gene expression (2 weeks; 6 weeks at very high vector dose)	[340,341]
-	rat tendon injury	mRNA (FGF-2)	effective gene expression, stronger mechanical properties (2 weeks)	[344]

Abbreviations: *lacZ*, β-galactosidase; PDGF, platelet-derived growth factor; ODN, oligodeoxyribonucleotide; mRNA, messenger ribonucleic acid; BMP-7, bone morphogenetic protein 7; FGF-2, basic fibroblast growth factor.

**Table 5:** Applications of classical gene therapy for indirect ACL repair ex vivo.

Vectors	Animal Models	Cells	Genes	Effects	Refs.
nonviral vectors	minipig ACL lesion	ACL graft	GFP, BMP-6	effective gene expression (2 weeks), ACL repair with stronger mechanical properties (8 weeks)	[346]
adenoviral vectors	mouse ectopic injection (muscle)	murine MSC line	BMP-12	high collagen deposition, neoligament formation (4 weeks)	[316]
	rabbit ligament lesion	rabbit ACL cells	*lacZ*	effective gene expression (2 weeks)	[341]
	rabbit ACL lesion	rabbit MSCs	TGF-β, VEGF	ACL remodeling with stronger mechanical properties (24 weeks)	[348]
		rabbit tendon graft	*lacZ*, BMP-2	effective gene expression (2 weeks), high collagen deposition with stronger mechanical properties (8 weeks)	[347]
retroviral vectors	rabbit ligament lesion	rabbit ACL cells	*lacZ*	effective gene expression (10 days)	[340]
		rabbit MSCs	PDGF	high collagen deposition, ligament remodeling (12 weeks)	[333]
	mouse tendon injury	human tendon-derived stem cells	lncRNA (H19)	high type-I collagen deposition, TNMD, decorin contents (4 weeks)	[334]
lentiviral vectors	rat ACL lesion	human ACL-derived stem cells	BMP-2	high type-I/-III collagen deposition, α-SMA contents, ACL repair (8 weeks)	[326]
		rat ACL-derived stem cells	VEGF	high type-I collagen deposition, ligament remodeling with stronger mechanical properties (4 weeks)	[325]
	rat tendon injury	rat tendon cells	shRNA (decorin)	high collagen deposition (4 weeks)	[335]

Abbreviations: ACL, anterior cruciate ligament; MSCs, mesenchymal stromal cells; GFP, green fluorescent protein; BMP, bone morphogenetic protein; *lacZ*, β-galactosidase; TGF-β, transforming growth factor beta; VEGF, vascular endothelial growth factor; PDGF, platelet-derived growth factor; lncRNA, long non-coding RNA (H19 involved in TGF-β signaling); shRNA, short hairpin RNA; α-SMA, alpha-smooth muscle actin.

**Table 6:** Applications of biomaterial-guided gene therapy for ACL repair.

Animal Models	Vectors	Cell Targets	Genes	Biomaterials	Effects	Refs.
-	nonviral vectors	chicken tendon cells	GFP, miRNA (TGF-β)	PLGA nanospheres	effective gene expression (GFP), suppression of TGF-β expression (miRNA TGF-β) (3 weeks)	[384]
	adenoviral vectors	bovine ACL cells	GFP	type-I collagen gel	effective gene expression (3 weeks)	[317]
		bovine ACL explants (torn)	TGF-β	type-I collagen gel	effective gene expression, high type-I/-III deposition, ACL repair (4 weeks)	[317]
		human ACL explants (torn)	GFP, IGF-I	type-I collagen gel	effective gene expression, high DNA contents (IGF-I) (3 weeks)	[318]
rabbit ACL lesion	nonviral vectors	-	TGF-β	tendon graft	high collagen deposition, wound healing with stronger mechanical properties (6 months)	[385]
chicken tendon injury	nonviral vectors	-	miRNA (TGF-β)	PLGA nanospheres, 3DB composite (PCL, PDA NPs, gelatin, HA, alginate)	suppression of TGF-β expression, wound healing (6 weeks)	[384,386]
rat tendon injury	adenoviral vectors	-	BMP-12	muscle graft	high collagen deposition, wound healing (4 weeks)	[319]
	lentiviral vectors	-	shRNA (decorin)	tendon graft	high collagen deposition, wound healing (4 weeks)	[335]
mouse tendon injury	rAAV vectors	-	*luc*, GDF-5	tendon graft	effective gene expression, wound healing with stronger mechanical properties (2–3 weeks)	[387,388]
rat ACL lesion, tendon injury	-	-	miRNA (angiogenic miR-210)	type-I collagen gel	high type-I collagen deposition, VEGF expression, wound healing with stronger mechanical properties (4–12 weeks)	[389,390,391]

Abbreviations: ACL, anterior cruciate ligament; rAAV, recombinant adeno-associated viral vectors; GFP, green fluorescent protein; miRNA, microRNA; TGF-β, transforming growth factor beta; IGF-I, insulin-like growth factor I; BMP-12, bone morphogenetic protein 12; shRNA, short hairpin RNA; GDF-5, growth differentiation factor 5; *luc*, luciferase; PLGA, polylactic-co-glycolic acid; PCL, polycaprolactone; PDA NPs, polydopamine nanoparticles; HA, hyaluronic acid; VEGF, vascular endothelial growth factor.

## Data Availability

Not applicable.
